# Regulated Assembly of LPS, Its Structural Alterations and Cellular Response to LPS Defects

**DOI:** 10.3390/ijms20020356

**Published:** 2019-01-16

**Authors:** Gracjana Klein, Satish Raina

**Affiliations:** Unit of Bacterial Genetics, Gdansk University of Technology, Narutowicza 11/12, 80-233 Gdansk, Poland

**Keywords:** LpxC, LapB, RpoE sigma factor, Rcs two-component system, lipid IV_A_, lipid A modifications, Lpt transport system, noncoding small regulatory RNA

## Abstract

Distinguishing feature of the outer membrane (OM) of Gram-negative bacteria is its asymmetry due to the presence of lipopolysaccharide (LPS) in the outer leaflet of the OM and phospholipids in the inner leaflet. Recent studies have revealed the existence of regulatory controls that ensure a balanced biosynthesis of LPS and phospholipids, both of which are essential for bacterial viability. LPS provides the essential permeability barrier function and act as a major virulence determinant. In *Escherichia coli*, more than 100 genes are required for LPS synthesis, its assembly at inner leaflet of the inner membrane (IM), extraction from the IM, translocation to the OM, and in its structural alterations in response to various environmental and stress signals. Although LPS are highly heterogeneous, they share common structural elements defining their most conserved hydrophobic lipid A part to which a core polysaccharide is attached, which is further extended in smooth bacteria by *O*-antigen. Defects or any imbalance in LPS biosynthesis cause major cellular defects, which elicit envelope responsive signal transduction controlled by RpoE sigma factor and two-component systems (TCS). RpoE regulon members and specific TCSs, including their non-coding arm, regulate incorporation of non-stoichiometric modifications of LPS, contributing to LPS heterogeneity and impacting antibiotic resistance.

## 1. Introduction

The cell envelope of Gram-negative bacteria, including *Escherichia coli*, contains two distinct membranes, an inner (IM) and an outer (OM) membrane separated by the periplasm, a hydrophilic compartment that includes a layer of peptidoglycan. The OM is an asymmetric bilayer with phospholipids forming the inner leaflet and lipopolysaccharide (LPS) forming the outer leaflet. LPS is essential for bacterial viability. Because of the strong lateral chemical interactions, LPS provides the essential permeability barrier function. It is a complex glycolipid, a major component of the OM and highly heterogeneous in composition. Bacteria, like *E. coli* and *Salmonella*, contain approximately 2-3 × 10^6^ molecules of LPS that cover more than 75% of the OM [1]. The biosynthesis, translocation and various modifications of LPS requires the function of more than 100 genes. Several of them are essential and unique to bacteria and hence they are excellent targets for the identification of their inhibitors for the development of new antibiotics.

The biosynthesis of LPS begins with the acylation of UDP-GlcNAc with *R*-3-hydroxymyristate derived from *R*-3-hydroxymyristoyl-ACP by LpxA [2]. *R*-3-Hydroxymyristoyl-ACP also serves as a precursor for the synthesis of phospholipids. The second reaction of the lipid A biosynthesis is catalysed by LpxC [UDP-3-*O*-(*R*-3-hydroxymyristoyl)-*N*-acetylglucosamine deacetylase] a Zn^2+^-dependent deacetylase, constituting the first committed step in the LPS synthesis, as the equilibrium constant for the first reaction catalysed by LpxA is unfavourable. Following deacetylation, a second *R*-3-hydroxymyristate chain is added by LpxD leading to the synthesis of UDP-2,3-diacyl-GlcN. This serves as a substrate for LpxH to generate 2,3-diacyl-GlcN-1-phosphate, also called lipid X [3]. The next steps involve a condensation reaction generating the β,1′-6-linked disaccharide by LpxB, followed by its phosphorylation at 4′ position by LpxH. This generates the lipid IV_A_ precursor, which serves as an acceptor for the WaaA-mediated incorporation of two 3-deoxy-α-d-*manno*-oct-2-ulsonic acid (Kdo) residues. Up to the synthesis of Kdo_2_-lipid IV_A_, all the required seven enzymes are essential for the bacterial viability [4]. Kdo_2_-lipid IV_A_ comprises a key intermediate in LPS biosynthesis that acts 2-fold as a specific substrate: (*i*) for acyltransferases that generate Kdo_2_-lipid A moiety by the transfer of two additional fatty acids to the (*R*)-3-hydroxyl groups of both acyl chains, which are directly bound to position 2′ and 3′ of the non-reducing GlcN residue and (*ii*) for glycosyltransferases catalyzing further steps of the core oligosaccharide biosynthesis. The *E. coli* K-12 genome encodes three paralogous acyltransferases (LpxL, LpxM and LpxP), which catalyze acylation reactions using acyl carrier protein-activated fatty acids as co-substrates [5]. At ambient temperatures, a lauroyl residue is first transferred by LpxL to the OH group of the amide-bound (*R*)-3-hydroxymyristate residue at position 2′. This catalytic step is partially replaced at low temperature (12 °C) by LpxP, which transfers palmitoleate to the same position in approximately 80% of LPS molecules [6]. The free OH group of the ester-bound (*R*)-3-hydroxymyristate residue at position 3′ within both pentaacylated intermediates is then myristoylated by LpxM to give a hexaacylated lipid A moiety. However, it is noteworthy that under slow growth conditions and at low temperatures, the lipid IV_A_ precursor without the Kdo incorporation can be the substrate for a secondary acylation by LpxP, LpxL, and LpxM, as observed in Δ*waaA* grown at 21–23 °C [7].

In *Enterobacteriaceae* members, the core region of LPS is always attached to lipid A via a Kdo residue. The inner core usually contains residue(s) of Kdo and l-*glycero*-d-*manno*-heptose (l,d-Hep) [4,8]. Several of lipid A biosynthesis enzymes and core biosynthetic glycosyltransferases are either inner membrane-anchored or membrane-associated and hence the LPS synthesis occurs at the IM leaflet. After the completion of LPS synthesis on the inner leaflet of the IM, LPS is flipped by the essential IM-located MsbA transporter to the periplasmic side of the IM, where it is a substrate for the LptB_2_FG ABC transporter for translocation, using ATP as the energy, potentially in complex with LapA/B proteins.

## 2. Regulatory Steps in LPS Biosynthesis

**Figure 1:**
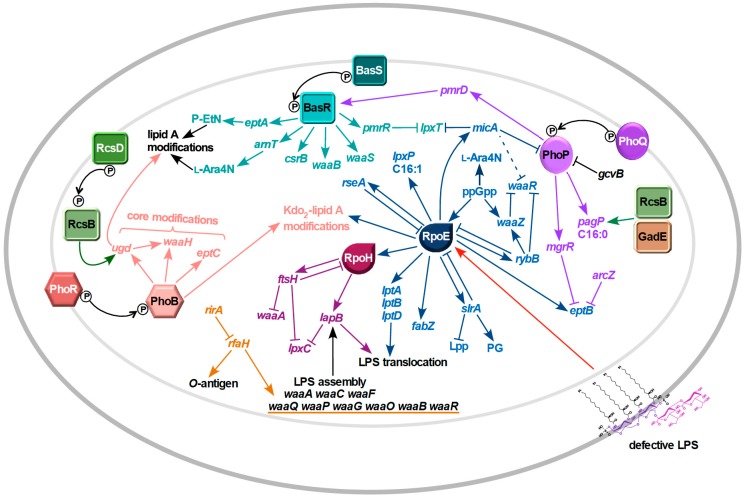
Networks of regulatory pathways that control the lipopolysaccharide (LPS) assembly and its non-stoichiometric modifications. The RpoE sigma factor responds to severe defects in LPS biosynthesis and is also required for transcription of genes involved in LPS biosynthesis/translocation and modifications via sRNAs like *rybB* and *micA*. RpoE also transcribes the *slrA* (*micL*) sRNA, which represses the Lpp synthesis and acts in a feedback manner to repress RpoE. Other regulatory controls involve two-component systems like BasS/R, PhoP/Q, PhoB/R and Rcs system, which are required for transcription of genes whose products are involved in lipid A and inner core modifications. The RpoH heat shock sigma factor transcribes *lapB* and *ftsH* genes, whose products control balanced biosynthesis of LPS and phospholipid by regulating LpxC levels. The unique transcriptional factor RfaH is required for overcoming antitermination, enhance transcriptional elongation and couple transcription/translation of *waaQ* and *rfb* LPS biosynthetic operons. The sRNA RirA binds to RfaH and abrogates its activity to maintain a balanced biosynthesis of LPS.

Until recent discoveries, it was presumed that LPS biosynthesis occurs in a constitutive manner. However, recent studies have revealed regulatory controls exerted right from early steps in LPS biosynthesis till its final delivery in the OM as highlighted below: (a) Regulation of GlmS expression for the synthesis of UDP-GlcNAc, an essential metabolic precursor for LPS and peptidoglycan, by recruiting GlmZ/Y noncoding small regulatory RNAs (sRNAs). (b) Balanced synthesis of phospholipids and LPS by regulated turnover of LpxC by FtsH/LapB proteins, since they use *R*-3-hydroxymyristoyl-ACP as a common precursor [9]. (c) Bacteria also ensure that only completely synthesized LPS is delivered to the Lpt translocation system by preferential selectivity of hexaacylated LPS by the MsbA transporter and by recruiting LapA and LapB proteins as a scaffold for various LPS biosynthetic enzymes in the IM. (d) The transcriptional control by RfaH of the large *waaQ* operon encoding various LPS core biosynthetic enzymes and the *rfb* operon whose products are required for *O*-antigen biosynthesis [10,11,12]. (e) Regulation of the incorporation of non-stoichiometric modifications of LPS by the induction of genes, whose expression is controlled by BasS/R, PhoP/Q and PhoB/R two-component systems (TCSs) (Figure 1). The expression of some of these genes is regulated either at a transcriptional or post-transcriptional level or both [13]. (f) Some of the genes, whose products are involved in either lipid A biosynthesis, LPS translocation or phospholipid biosynthesis, are transcriptionally regulated by the RpoE sigma factor and the CpxA/R TCS [14]. (g) The LPS assembly in the OM requires a correctly folded outer membrane protein LptD. Its correct folding is SurA- and DsbA-dependent, which can be further fine-tuned by BepA for the removal of misfolded LptD.

## 3. Essentiality of LPS and the Minimal LPS Structure

Generally, LPS is essential for the viability of vast majority of Gram-negative bacteria with few exceptions. Few limited exceptions include viable LPS-lacking mutants of *Acinetobacter baumannii* that have mutations in either *lpxA* or *lpxC* or *lpxD* genes [15]. However, in such mutant strains, the absence of LPS is compensated by increased expression of the Lol lipoprotein transport system to increase phospholipid export, enhanced expression of poly-β-1,6-*N*-acetylglucosamine and elevated expression Mla-retrograde phospholipid migration from the OM to the IM to maintain a balance in the essential constituents of the OM [15]. Another well-studied example of dispensability of LPS include construction of viable LPS deficient mutants of *Neisseria meningitides* [16].

It is well established that in bacteria, like *E. coli* and *Salmonella*, the minimal structure of LPS that can sustain the bacterial viability up to 42 °C is composed of hexaacylated lipid A-Kdo_2_ (Re LPS “deep-rough mutants”). Thus, Δ*waaC* (lacking heptosyltransferase I) or Δ*gmhD* (absence of ADP-l-*glycero*-d-*manno*-heptose-6-epimerase) mutants are viable, although they exhibit temperature sensitive growth (unable to grow at temperatures above 43 °C), permeability defects, hypersensitivity to detergents, hyperactivated RpoE-regulated stress response, inability to colonize the host, sensitivity to antimicrobial peptides, defects in flagellar biosynthesis, compromised growth at low pH, and a constitutive induction of Rcs-dependent exopolysaccharide [7,17,18,19,20,21]. Consistent with the requirement of inner core heptose attachment to Kdo_2_-lipid A for cell envelope integrity, a Δ(*waaC surA*) mutational combination confers synthetic lethality [7]. SurA is a major periplasmic folding factor required for the folding of outer membrane proteins (OMPs), including LptD [17,22]. Furthermore, the lack of other conserved core biosynthetic genes like *waaG*, *waaF* or genes involved in the pathway of synthesis of molecules of l-*glycero*-α-d-*manno*-heptose (heptose) such as *gmhA* and *gmhE*, also results in impairment of growth at high temperatures [23]. A deep-rough phenotype accompanied by hypersensitivity to detergents and antibiotics is also associated with the lack of WaaP kinase, which mediates phosphorylation of HepI and completion of core biosynthesis [24] (Figure 1). Underacylation of lipid A due to a lack of the LpxL lauroyl transferase is known to confer temperature sensitivity above 33 °C and strains lacking all three acyltransferases Δ(*lpxL lpxP lpxM*) cannot grow at even 30 °C on rich medium [5]. Moreover, Δ(*lpxL lpxP lpxM*) strains exhibit gross alterations in terms of accumulation of primarily glycoform IV/V with three Kdo residues and truncation of the terminal disaccharide [7]. Thus, complete synthesis of LPS is a requirement for bacterial fitness and survival.

During the detailed genetic construction of strains to define the minimal LPS structure in the absence of any suppressors, it was shown that strains with LPS composed of either Kdo_2_-lipid IV_A_ Δ(*waaC lpxL lpxM lpxP*) or only lipid IV_A_ (absence of WaaA Kdo transferase) can be constructed under slow growth conditions on minimal medium at low temperatures (21–23 °C) [7]. Since lipid IV_A_ is a poor substrate for the MsbA IM LPS flippase, suppressor mutations that improved growth characteristics of Δ(*waaC lpxL lpxM lpxP*) or Δ*waaA* were found to map to the *msbA* gene [7]. It is likely that such MsbA variants exhibit altered binding properties of lipid A and might be more relaxed in substrate selectivity or changes in ATP binding/hydrolysis. Interestingly, Δ*waaA* suppressor-free strains synthesizing lipid IV_A_ were found to accumulate excess of phospholipids consistent with a balanced synthesis of LPS and phospholipids [7]. Moreover, such Δ*waaA* strains under slow growth conditions were also found to accumulate pentaacylated and hexaacylated species of lipid IV_A_ without any requirement for the Kdo presence. Thus, lipid IV_A_ derivatives with myristoyl, lauroyl, palmitolyl or palmitoleate chains could be identified from Δ*waaA* strains, indicating that under such conditions late acyltransferases can use lipid IV_A_ as a precursor without requirement for Kdo [7]. Consistent with these results, overexpression of the *lpxL* gene suppress the lethality of *waaA* deletions on nutrient broth up to 37 °C without the need for MsbA overproduction [25].

## 4. Regulation of Synthesis of UDP-GlcNAc-Precursor for LPS Biosynthesis

UDP-*N*-acetyl-d-glucosamine (UDP-GlcNAc) is a common metabolic precursor for LPS and peptidoglycan synthesis. Thus, regulation of biosynthesis of UDP-GlcNAc serves as an essential branch point in controlling and coupling the synthesis of major essential constituents of the cell envelope [13]. GlmS catalyzes the synthesis of glucosamine-6-phosphate (GlcN6P) from fructose-6-phosphate and glutamine, which constitutes the first committed step in the synthesis of UDP-GlcNAc. The amount of *glmS* transcript is regulated by a feedback mechanism in response to the GlcN6P level using homologous GlmZ and GlmY sRNAs [26,27]. These sRNAs act in a hierarchical manner to activate the *glmS* expression. Under GlcN6P limiting conditions, the GlmY sRNA accumulates and sequesters RNase adaptor protein RapZ, preventing GlmZ processing [26,27]. The GlmZ sRNA facilitates translation of the *glmS* mRNA through an anti-antisense mechanism and prevents the formation of an inhibitory structure that occludes the ribosome-binding site of *glmS*. Interestingly, transcription of the *glmY* gene is regulated by RpoN and RpoD sigma factors using the same transcription start site in an analogous manner to the transcriptional regulation of *rpoE*P2 and *rpoE*P3 promoters [11,28]. RpoN-regulated promoters of *glmY* and *rpoE* genes use QseF as an activator and thus this mode of regulation may be important to sense common signals and ensure cellular homeostasis in response to envelope stress.

## 5. Coupled Regulation of LPS and Phospholipids–Regulation of Amounts of Kdo_2_-Lipid A Synthesis

Regulation of LpxC occurs by regulated proteolysis mediated by FtsH [29,30]. This proteolysis by FtsH requires the LPS assembly factor LapB [9,31]. Both FtsH and LapB are essential for bacterial growth and their depletion causes increased synthesis of LPS at the expense of phospholipids. This is due to stabilization of LpxC in either an *ftsH* or a *lapB* mutant, which causes diversion of a common precursor *R*-3-hydroxymyristoyl-ACP towards the LPS synthesis, since LpxC and FabZ compete for the same precursor, limiting the availability of phospholipids (Figure 1). Consistent with the notion of coupling of phospholipid and LPS synthesis, suppressors mapping to the *fabZ* gene, like *sfhC*21 that encodes a hyperactive variant of FabZ, can bypass the essentiality of either *ftsH* or *lapB* genes [9]. Additional evidence supporting this model is based on observations that inhibition of LpxC can be compensated by mutations that compromise the FabZ activity [32]. Similarly, overexpression of the *fabZ* gene is accompanied by an upregulation of the LpxC activity and vice versa [32]. Further supporting regulated LPS and phospholipid biosynthesis, an overexpression of noncoding sRNA *slrA* can bypass the lethality of the essential *lapB* gene [9]. The molecular basis of this suppression was attributed to translational repression of the gene encoding the most abundant protein Lpp, also called Braun’s lipoprotein, with an abundance of 7 × 10^5^ molecules per cell. Hence, SlrA is also called MicL [33]. Each Lpp molecule has three acyl chains {phosphatidylglycerol moieties (PG)} and therefore, when the Lpp amount is reduced due to overexpression of *slrA* sRNA, it causes an increase in the amount of PG that can restore a balance between phospholipids and LPS (Figure 1). SlrA can also act as a negative regulator of RpoE in a feedback manner, as its overproduction reduces the RpoE activity elevated due to LPS defects in Δ*lapB* mutants [9]. The gene encoding SlrA sRNA is transcribed from the RpoE-regulated promoter located within the *cutC* gene. This 80-nt sRNA is synthesized as a 307 nt precursor mRNA that is processed and is located within the 3′ end of the coding region of the *cutC* gene [9,33].

Although a role for LapB and FtsH for LpxC is now known, however how the proteolytic activity of FtsH is regulated remains to be elucidated. It is likely that proteolytic activity of FtsH might be regulated by concentration or forms of acyl-ACPs or lipid A disaccharide. Since LpxC stability is increased in a *fabI*(ts) mutant suggests that membrane fatty acids might influence proteolytic activity or add additional checkpoints in regulating lipid A and phospholipid amounts [29]. Although not fully elaborated, regulation at the level of LpxB and LpxK may serve as additional pathways of this co-regulation. Consistent with such a notion, LpxB has been shown earlier to co-purify with phospholipids [34]. A regulatory checkpoint has been postulated on the basis of a reduced LpxK activity upon reduced membrane fluidity when more saturated fatty acids are present [35]. LpxK mediates the last essential step that completes lipid IV_A_ biosynthesis [4]. LpxK catalyzes the phosphorylation of the 4′ hydroxyl of the distal glucosoamine of lipid A disaccharide. Under conditions like compromised function of either LapB or FtsH, which stabilize LpxC leads to depletion of *R*-3-hydroxymyristoyl-ACP. This in turn decreases the synthesis of unsaturated fatty acids, which can decrease the LpxK activity, leading to accumulation of lipid A disaccharide [9,35]. This accumulation of lipid A disaccharide intermediate by the feedback mechanism causes increased proteolysis of LpxC, thereby decreasing flux of *R*-3-hydroxymyristoyl-ACP into a lipid A biosynthesis pathway, thus again to regain a balance between phospholipid and lipid A biosynthesis. WaaA is also one of the substrates of FtsH protease [36]. Regulation of WaaA turnover by FtsH may be yet another step in preventing an excessive synthesis of LPS over phospholipids. However, a direct impact of changes in WaaA concentration on either LPS synthesis or accumulation of different glycoforms remains to be understood.

## 6. Assembly of LPS Requires LapB

Besides a role for turnover of LpxC in concert with FtsH, LapB has been implicated in the assembly of LPS at the IM presumably along with LapA [9]. Genes encoding *lapA* and *lapB* are co-transcribed from three promoters, the distal promoter located upstream of the *pgsB* gene, the middle promoter recognized by the RpoH heat shock sigma factor and the last one resembling house-keeping promoters [9]. Overall, such a transcriptional organization suggests coupling of transcription with phospholipid metabolism using *pgpB* co-transcription. PgpB encodes phosphatidylglycerophosphatase, an enzyme that is part of the phosphatidylglycerol biosynthesis pathway, which is itself part of phospholipid metabolism. Transcription from the heat shock promoter ensures transcription of *lapA/B* genes at high temperatures, hence belonging to heat shock regulon, which comprises several chaperones and proteases.

The evidence that LapB plays a role for LPS assembly in the IM comes from experimental evidence that include: (a) The *lapB* gene is essential for bacterial growth and a deletion of the *lapB* gene can be constructed in the presence of suppressor that either restore phospholipid synthesis like *sfhC21*, or decrease the LPS synthesis like in the presence of mutations in an early lipid A biosynthesis pathway (*lpxA*, *lpxC*, *lpxD*) or when LPS is composed mostly of Kdo_2_-lipid A derivatives like (*waaC lapB*) combination [9]. (b) The lack of LapB leads to accumulation of LPS precursor forms. These precursor forms represent pentaacylated lipid A species, the presence of Kdo_2_-dilauroyl-lipid IV_A_. (c) Δ*lapB* mutants have defects in the folding of LPS-specific enzymes like LpxM and conserved glycosyltransferases like WaaC, WaaO. A large proportion of these enzymes is present in aggregate form and hence LPS-specific enzymes like LpxM and core glycosyltransferase could be limiting explaining the accumulation of LPS precursor species [9]. (d) LapB also co-purifies with heptosyltransferase I. (e) LapA/B could function synergistically with classical chaperones like DnaK/DnaJ based on LapA/LapB co-purification with DnaK/J, multicopy suppression of growth defects of Δ(*lap lapB*) mutants, even more pronounced defects in the LPS composition when Δ(*lapA lapB dnaK/J*) were examined. (f) LapA/B co-purify with LPS, Lpt proteins and FtsH. (g) A Δ(*lapA-lapB*) mutation is synthetically lethal with a compromised LptD variant or when SurA is absent. (h) The absence of LapB induces a strong envelope stress response regulated by RpoE, Cpx and Rcs systems by inducing transcription of genes, whose products are required to maintain homeostasis in the cell envelope. LapB contains nine tetratricopeptide repeat (TPR) motifs and a C-terminal rubredoxin domain [9,37]. Mutations in the either rubredoxin domain or TPR/interface impair cell growth [9]. Thus, based on the essentiality of TPR repeats in LapB, which can mediate protein-protein interactions (interaction of LapA/B with LPS-specific enzymes) a model was presented wherein LapA/B could form a scaffold-like structure for delivery of various acyltransferases and glycosyltransferases to the site in IM where LPS is assembled and delivered to the Lpt complex. At such an assembly site, proteolysis of LpxC can occur given co-purification of LapA/B with FtsH to prevent excessive LPS buildup. This may serve as an essential purpose of preventing wasteful transfer of incompletely synthesized LPS. This model draws support from the IM association of LpxC in *Neisseria meningitidis*, which requires the presence of LapB counterpart called Ght [38]. Consistent with a broad essential function of LapB, a suppressor mutation *lapB*V43G in the *lapB* gene that confers protection against prolonged exposure to phosphate starvation and also suppresses the *mlaA*-dependent hyperproduction of LPS have been reported [39,40]. Thus, LapB plays an important role in maintaining cell envelope homeostasis and assembly of LPS.

## 7. Transport of LPS

### 7.1. MsbA-Mediated Transport of LPS Across the Inner Membrane

After the completion of core-lipid A synthesis, LPS is flipped across the IM by the essential ATP-binding cassette MsbA transporter. Several structures have been determined, which show that MsbA exists as a functional dimer, wherein core-lipid A binds the cytoplasmic open conformation [41,42]. Upon ATP binding, conformational change in transmembrane (TM) domains results into change from the cytoplasmic open to the periplasmic open state causing flipping of core-lipid A into the periplasmic leaflet of the IM. MsbA can return to the cytoplasmic open state after release of phosphate [42]. MsbA has a higher preference for hexaacylated lipid A derivatives, thereby providing an early checkpoint to prevent transport of early intermediates of LPS biosynthesis. Recent structural analysis of MsbA-core lipid A revealed that MsbA recognizes a bivalent phosphoglucosamine headgroup and correct acylation of lipid A to achieve substrate selectivity over competing bulk membrane phospholipids [43]. The tight packing of hexaacaylated LPS in the hydrophobic pocket suggests that MsbA packs acyl chains of correct length and number, providing the basis of this selectivity [43].

### 7.2. LPS Translocation and Assembly in the Outer Membrane

After the flipping of LPS by MsbA to the periplasmic side of IM, LPS is transported for its final localization in the OM which requires a complex of seven essential conserved proteins LptA-LptG, whose components reside in every cell compartment and form a single transenvelope complex spanning from the IM to the OM. Here, we briefly summarise key elements, as this topic has been aptly reviewed recently [44,45,46,47]. The Lpt system is organized into the IM complex comprised of LptB_2_CFG and the OM component of 1:1 complex of LptDE, which are bridged by a periplasmic protein component LptA. This transenvelope complex acts as a single unit to transport LPS, since depletion of any component leads to LPS accumulation at the IM [44,45,46,47]. Mechanistically, the ABC transporter LptB_2_FG extracts LPS from the IM in an ATP-dependent manner to deliver to LptC, and LPS transfer from LptC to LptA requires additional ATP hydrolysis [44,45,46,47], allowing LPS transit across the aqueous periplasm for its final delivery to LptD. The evidence from co-sedimentation, pull-down experiments, photo-cross-linking, mutational and structural analyses, assembly of liposomes from IM components and OM components in the presence or absence of LptA, shows that, while the N-terminus of LptA interacts with LptC at the IM, the C-terminus of LptA interacts with the periplasmic domain of the OM LptD, creating a continuous bridge of antiparallel β-strands between the IM and the OM [44,45,46,47]. The presence of β-jellyroll domains in LptG, LptF, LptC, LptA and LptD can sequester the acyl chains of LPS from the aqueous periplasm, using ATPase activity of LptB to transfer LPS over transenvelope bridge [48]. In support of the single stable bridge model of LPS transport, it was shown that LptA can promote association of liposomes containing the IM complex of LptBCFG with OM liposomes containing the OM LptDE [49]. Furthermore, ATP-dependent transfer of LPS from LptBFG to LptC and LptA has been demonstrated in liposomes and transfer to LptA was shown to be increased when LptC was present [49].

Structural studies of LptD and LptE revealed that LptD folds into two domains: a β-jellyroll and a β-barrel [50,51]. The β-jellyroll extends away from the OM and interacts with the β-jellyroll domain of LptA suitable for binding to lipid A, while leaving the LPS oligosaccharide exposed. LPS could be delivered directly into the cavity of the larger domain of LptD β-barrel, which is composed of 26 membrane-spanning β-strands. LptE adopts a roll-like structure located inside the barrel of LptD to form a unique two-protein ‘barrel and plug’ architecture [50,51]. The plugging of LptE inside of the LptD barrel causes a diminished lumen size to 45Å × 35Å on the periplasmic side. However, such a space is sufficient to accommodate LPS. In these structures, β-strands 1 and 2 are distorted and weak hydrogen bonding between β-strands 1 and 26 could support a lateral opening for LPS migration. In this process, positioning of the lipid A part could be assisted by LptE, as it is known to bind LPS and oligosaccharide could be placed in the hydrophilic cavity of LptD chamber.

Finally, LptD folding into the functional state can be a rate-limiting step. Several periplasmic folding catalysts like DsbA, DsbC, SurA, FkpA are known to play important roles in the folding of OMPs and periplasmic proteins [52]. Folding of LptD requires the SurA periplasmic folding catalyst and other periplasmic folding factors, like Skp and FkpA, could further modulate efficient folding of LptD [22]. LptD has two disulfide bridges between four non-consecutive cysteine residues, which require the periplasmic DsbA disulfide oxidoreductase and interaction of LptD with LptE to achieve correct folding with native disulfide bridges [53]. Additional factors like BepA may also be required for stimulating disulfide rearrangement of LptD and degradation of misfolded LptD [54]. From the transcriptional point of view, the *lptD* gene is transcribed by Eσ^E^ [14] and this discovery was the beginning of series of studies that led to subsequent elucidation of the Lpt system.

## 8. Regulated Structural Alterations in LPS

The LPS composition is highly heterogeneous and dynamically altered in response to various challenges like exposure to different stress conditions or changes in growth medium. Heterogeneity of LPS can be due to: modification of the lipid A part by the incorporation of phosphoethanolamine (P-EtN) and 4-amino-4-deoxy-l-arabinose (l-Ara4N) that mask negative charges, the addition or removal of acyl chains, changes in the inner core due to the incorporation of additional Kdo residue, rhamnose (Rha), uronic acid and P-EtN and changes in number of phosphate residues, and truncation of the outer core [55]. All these structural changes are highly regulated, important for resistance to cationic antimicrobial peptides, for bacterial virulence in pathogenic bacteria and may have adaptive significance in specific environmental niches. Certain modifications in the LPS structure can also contribute to biofilm tolerance of antimicrobial compounds [56]. Lipid A of *E. coli* under standard growth conditions is bisphosphorylated carbohydrate backbone disaccharide β-d-Glc*p*N4P-(1→6)-α-d-Glc*p*N1P, which is hexaacylated without any modifications. However, exposure to low pH, excess of Fe^3+^, Zn^2+^, Al^3+^, change in divalent cations concentrations, challenge by antimicrobial peptides, treatment with the non-specific phosphatase inhibitor ammonium metavanadate (AMV), treatment with agents that disturb the OM symmetry like exposure to chelating agents like EDTA or genetic alterations that lead to the synthesis of tetraacylated derivatives can cause profound changes in the lipid A composition [7,55,57]. Some of these modifications are regulated at the transcriptional level, while some are subjected to a post-transcriptional control and certain modification occur at post-translational level [13].

### 8.1. Regulation of Lipid A Modifications

Most prevalent non-stoichiometric modifications that occur in the lipid A part involve either reducing the net negative charges of lipid A by modifying the 1 and/or 4′ ends of phosphate residues, or the addition or removal of acyl chains. Most commonly observed modifications of lipid A include the incorporation of P-EtN and l-Ara4N at 1 and/or 4′ ends, respectively [57]. Such substitutions are known to confer resistance to cationic antimicrobial peptides like polymyxin B. This non-stoichiometric incorporation of P-EtN and l-Ara4N residues requires IM-located EptA and ArnT transferases, respectively with active site facing the periplasm [57]. Genes encoding these transferases are part of operons, whose transcription is positively regulated by the BasS/R (PmrA/B) TCS with an overlap with the PhoP/Q system. These systems specifically respond to changes in Fe^3+^ and divalent cationic concentrations, respectively. The PhoP/Q system in *Salmonella* and in some pathogenic Gram-negative bacteria regulates the expression of virulence genes, including those encoded in pathogenic islands. Lipid A of strains synthesizing tetraacylated lipid A can have phosphate residues at 1 and/or 4′ ends modified by P-EtN, since they do not incorporate l-Ara4N due to defects in translocation and such lipid A species could be poor substrates for ArnT. Such modifications occur after the translocation at the periplasmic side and often serve as markers for LPS translocation with l-Ara4N incorporation as a more stringent signature [7]. In *E. coli* and *Salmonella,* the activation of PhoP/Q TCS upon depletion of Mg^2+^ and Ca^2+^ also leads to the BasS/R induction, which requires PmrD as the adaptor protein [58]. Such a cross talk between BasS/R and PhoP/Q TCSs allows integration of signals from different environmental cues and amplification of output response.

The majority of lipid A in *E. coli* K-12 contains monophosphate at positions 1 and 4′. Approximately one-third of lipid A molecules in the *E. coli* K-12 outer membrane contains a diphosphate unit at the 1 position. The enzyme LpxT is responsible for this phosphorylation at 1′ position, generating hexaacylated lipid A with two phosphate residues under ambient growth conditions in the absence of induction of lipid A modification systems increasing the negative charge [59]. However, upon induction of the BasS/R system, P-EtN is incorporated at 1′ position due to inhibition of the LpxT activity. This inhibition of LpxT activity upon BasS/R-inducing conditions is due to the expression of a short peptide PmrR, which directly bind to LpxT [60] and hence constitutes a post-translational control [13]. In *Salmonella*, PhoP activation can also positively regulate *lpxT* transcription. The PhoP-dependent *lpxT* expression induced in low Mg^2+^ results in 1-PP lipid A, favors further modification of lipid A phosphates with l-Ara4N and the exclusion of P-EtN. Thus, *Salmonella* favors lipid A modified with l-Ara4N under low Mg^2+^ and with both l-Ara4N and P-EtN when exposed to a mildly acidic pH [61].

As RpoE and LPS structural alterations are intricately linked, an interesting regulatory control of lipid A alteration by RpoE-regulated sRNAs has emerged. Structural analysis of LPS obtained from several different strains under simultaneous RpoE- and BasS/R-inducing conditions shows the absence of LpxT-dependent phosphorylation of lipid A, presumably due to the transcriptional induction of *micA* [62]. The RpoE-regulated MicA sRNA can also exert an influence on lipid A composition by connecting regulation of PhoP/Q TCS as well as LpxT to RpoE. Analysis of MicA targets revealed that besides known OMP-encoding genes, MicA represses PhoP synthesis by base pairing in the translation initiation region of *phoP* mRNA and inhibits its translation [63]. MicA sRNA could as well regulate the LpxT synthesis at a post-transcriptional level, since a base-paring region between the seed sequence in the *micA* sRNA and the *lpxT* mRNA has been predicted [64]. Another sRNA GcvB also represses *phoP* mRNA translation by base-pairing [65].

The lipid A part can also undergo non-stoichiometric modification by PagP-dependent palmitoylation due to the incorporation of a palmitate chain linked to the hydroxyl group of 3-hydroxymyristic acid at C-2 position of reducing-end [66]. Transcription of the *pagP* gene is positively regulated by the PhoP/Q TCS and via RcsB in biofilm environment or increase in osmolarity that requires the GadE auxiliary regulator independently of the Rcs phosphorelay cascade [56]. The PagP enzyme is usually inactive in the OM and is post-transcriptionally activated when the OM permeability is breached and this modification can dampen recognition by host immune responses. Another PhoP/Q-regulated modification is PagL-dependent deacylation. The *pagL* gene encodes a lipid A 3-*O*-deacylase and this deacylation of lipid A modifies its ability to induce immune response [67]. The PagL-dependent modification also occurs post-translationally after LPS is incorporated in the OM [13]. It is noteworthy that in *Pseudomonas aeruginosa* the expression of *pagL* is positively regulated by sRNA (Sr006) [68]. Regulation of another deacylase LpxR with a 3′-*O*-deacylase activity of relevance to pathogenicity is of interest and this modification also occurs in the OM [69,70]. The expression of LpxR is subjected to negative regulation by MicF *trans*-acting base-pairing RNA. The base-pairing of MicF within the coding sequence of the *lpxR* mRNA decreases its stability by promoting its degradation by RNase E [71]. Thus, variety of mechanisms exist to modify lipid A part of LPS which involve TCSs, the RpoE sigma factor and sRNAs and this regulation can occur at different steps of LPS biosynthesis transcriptionally or post-transcriptionally.

### 8.2. Regulation of Inner Core Modifications and Switches Between Different Glycoforms

The inner core of LPS in the majority of cases generally contains an α-(2-4)-linked Kdo disaccharide, to which l-*glycero*-d-*manno*-heptose residue (Hep) is attached at position 5 of KdoI. In *E. coli*, the inner core contains three Hep residues. Although the composition of the inner core is relatively conserved, Kdo, as well as Hep residues, can be non-stoichiometrically modified. These modifications are regulated by the RpoE sigma factor, PhoP/Q, PhoB/R TCSs, by alarmone ppGpp and recruitment of specific sRNAs, whose expression is regulated by these transcription factors (Figure 1). Like lipid A modifications, they are often important for antibiotic resistance OM permeability and contribute to diversity in the LPS composition [10,13,62]. Quite well studied modifications include the non-stoichiometric incorporation of a P-EtN residue on the second Kdo by EptB transferase, incorporation of a third Kdo by WaaZ transferase, the addition of Rha, which can be linked to either the second Kdo or the third Kdo, modification of phosphorylated HepI by P-EtN using the PhoB/R-regulated EptC and incorporation of glucuronic acid (GlcUA) by the PhoB/R-inducible WaaH glycosyltransferase with a concomitant loss of phosphate residue on HepII [55,62].

Transcription of the *eptB* gene encoding P-EtN transferase specific to the second Kdo is positively regulated by the RpoE sigma factor [7,72]. Hence, this modification of the second Kdo is quite pronounced when the RpoE activity is induced either in the absence of RseA anti-sigma factor or when LPS is defective as in Δ*waaC* or Δ*waaF* mutants [7,11,62]. Consistent with the induction of RpoE and regulation of the *eptB* transcription, the lipid A part of Δ*waaC* or Δ*waaF* strains were found to lack P-EtN even under *eptA*-inducing conditions, but preferentially incorporating P-EtN on the second Kdo [7,11]. However, this incorporation requires the presence of Ca^2+^ in the growth medium. Furthermore, the synthesis of EptB is negatively regulated by two Hfq-dependent sRNAs MgrR and ArcZ, although in response to different environmental signals [72,73]. Under normal growth conditions, the PhoP-regulated MgrR sRNA via base-pairing with the *eptB* mRNA silences its expression due to translational repression of the *eptB* mRNA. Activation of PhoP/Q induces transcription of the *mgrR* sRNA. The EptB-dependent P-EtN modification of the second Kdo upon high Ca^2+^ concentration can be explained by the repression of transcription of PhoP/Q-regulated *mgrR* and promote the synthesis of active EptB under RpoE-inducing conditions. The Hfq-dependent ArcZ sRNA also inhibits translation and expression of *eptB* by base-pairing in an ArcA/B-dependent manner in response to oxygen concentration [72]. Quite like the P-EtN modification of lipid A, the incorporation of P-EtN on the second Kdo confers resistance to polymyxin B. Thus, RpoE regulon members like *eptB* and MicA sRNA, PhoP-regulated MgrR sRNA contribute to the incorporation of P-EtN on the second Kdo, lipid A and regulation of glycoform switches.

### 8.3. Modifications in the Heptose Region of the LPS Inner Core

**Figure 2:**
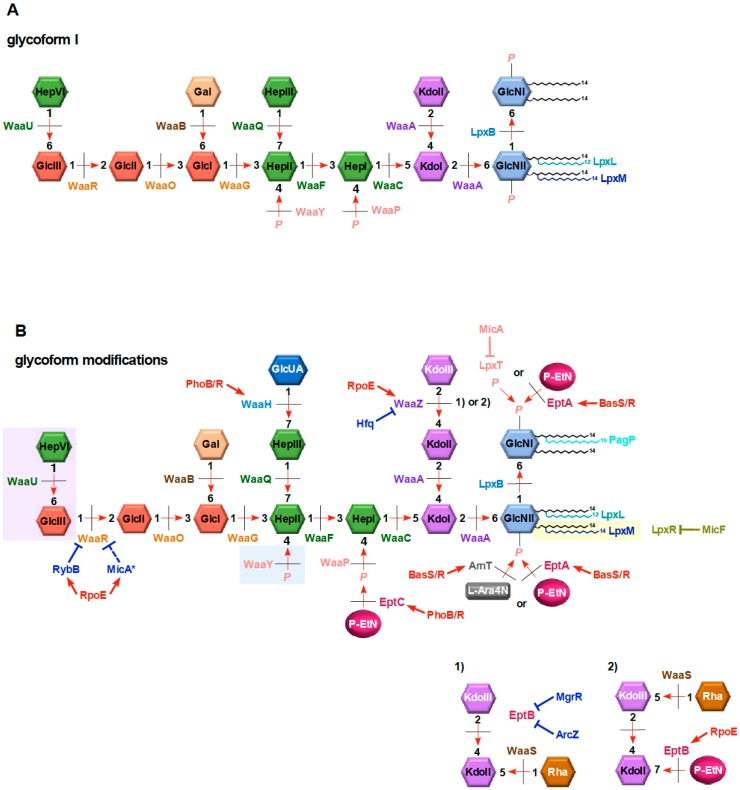
Schematic depiction of unmodified hexaacylated glycoform I and glycoform derivatives with various non-stoichiometric substitutions with a third Kdo-Rha disaccharide. Various genes whose products mediate different steps in LPS biosynthesis and incorporation of different modifications are indicated. Glycoform I constitutes the major LPS species under non-stress conditions (**A**). Upon the induction of RpoE sigma factor and conditions inducing BasS/R and PhoB/R TCSs, the lipid A part is modified by P-EtN and l-Ara4N at 1 and 4′ position by EptA and ArnT, respectively (**B**). The induction of RpoE causes increased synthesis of EptB overcoming MgrR silencing and incorporation of P-EtN on the second Kdo (panels B1 and B2). The induction of RpoE favors pathway of the third Kdo incorporation by increased synthesis of WaaZ and repression of WaaR by the RybB sRNA, leading to synthesis of glycoforms with a third Kdo with attachment of Rha on the third Kdo with a concomitant truncation of the terminal GlcIII-HepIV disaccharide shown in violet background. The PhoB/R induction leads to the incorporation of GlcUA at the expense of HepII phosphorylation (blue background). Lipid A can also be modified by PagP generating heptaacylated lipid A and by the removal of acyl chains by LpxR in the OM after translocation (light brown background). Various sRNA-mediated controls are shown in blue color.

The inner core of LPS exhibits the limited structural diversity, since it plays a crucial role in maintaining the OM stability. However, some non-stoichiometric substitutions have been demonstrated in this region of LPS. These include the incorporation or loss of phosphate residues and the addition of P-EtN, GlcN, or GlcUA. Among these, the phosphorylation of the HepI residue by WaaP is critical for the OM permeability and also provides the attachment sites for the other substituents and ensures the completion of core synthesis [11,24,55]. Accordingly, in *E. coli* K-12, phosphorylated HepI serves as an acceptor for P-EtN, which requires the EptC phosphoethanolamine transferase [55]. Furthermore, the HepIII residue, whose incorporation requires prior phosphorylation of HepI by WaaP, can be modified by GlcN (in *E. coli* R1 and R3 isolates) or by GlcUA (in *Salmonella* and *E. coli* B, K-12, R2 and R4 core types). Importantly, the modification of the HepIII residue is always accompanied by loss of phosphate residue at the HepII, thereby maintaining a net negative charge [55,74]. Structural studies revealed that GlcUA is attached to O-7 of the side-chain Hep, with the phosphate residue found at position O-4 of the HepII being absent [55] (Figure 2). WaaH shares 20% amino acid sequence similarity with WabO of *Klebsiella pneumoniae*. However, WabO in *K. pneumoniae* is responsible for the galacturonic acid (GalA) incorporation [75]. In *E. coli* K-12, *waaH* and *eptC* genes, whose products mediate transfer of GlcUA and P-EtN modifications of the Hep I and Hep III residues respectively, are positively regulated by the PhoB/R TCS [55]. However, the P-EtN-modification of HepI can also occur at the basal level without a requirement for induction of either PhoB/R or BasS/R systems. The EptC-dependent modification of HepI is important for the permeability function, as Δ*eptC* mutants exhibit sensitivity to exposure to sub-lethal Zn^2+^ concentration or the presence of SDS [55].

### 8.4. Glycoform Switches

Up to now, at least seven structurally different glycoforms of *E. coli* K-12 have been characterized, whose relative abundance varies on growth conditions such as exposure to phosphate-limiting growth conditions, the induction of RpoE-regulated envelope stress response and activation of specific TCSs. These glycoforms differ due to the non-stoichiometric modifications such as P-EtN transfer to the second Kdo, incorporation of a third Kdo, addition of sugars like Rha, GlcN, uronic acids, alterations in the numbers of phosphate residues in the LPS core and truncation of outer core terminal disaccharide. In initial studies using optimal growth conditions, the wild-type *E. coli* K-12 was found to contain majority of LPS corresponding to glycoform I structure and only minor amounts of three additional glycoforms II, III and IV could be observed [76]. Glycoform I contains two Kdo residues in the inner core, and four heptoses and four hexoses attached in specific order in the inner core and the outer core (Figure 2). However, using strains or growth conditions that exhibit the induction of RpoE sigma factor or employing phosphate-limiting growth conditions supplemented with Fe^3+^ and Zn^2+^ (induction of PhoB/R and BasS/R TCSs) or treatment with AMV (induction of RpoE and non-specific induction of TCSs) revealed major shifts in the LPS composition and prevalence of different glycoforms not only in *E. coli* K-12 but also in *E. coli* strains with different core type and in *Salmonella* [55,62]. Using growth medium that induces BasS/R and PhoB/R TCSs, wild-type *E. coli* strains synthesize more glycoform IV/V derivatives as compared to glycoform I. However, when the RpoE induction is maximal, a near-exclusive synthesis of glycoform V and its derivatives are observed (Figure 2). This molecular switch to the synthesis of glycoform V derivatives requires ppGpp alarmone, induction of the RpoE-transcribed genes *eptB*, sRNAs *micA* and *rybB*, and the transcriptional upregulation of *waaZ* with a concomitant repression of WaaR synthesis [62] (Figure 2). *waaZ* and *waaS* genes encode the Kdo transferase required for the incorporation of the third Kdo and rhamnosyl transferase, respectively, while as the *eptB* gene encodes P-EtN transferase specific to the second Kdo [62,77] (Figure 2). Glycoforms IV and V have the same molecular masses, but are structurally different due to the incorporation of P-EtN on the second Kdo by the RpoE-regulated EptB and attachment of Rha to the terminal third Kdo defining glycoform V. Without RpoE induction, EptB synthesis is silenced by the PhoP/Q-dependent MgrR sRNA (no incorporation of P-EtN on the second Kdo, instead the attachment of Rha on the second Kdo) and hence the synthesis of glycofom IV derivatives.

One of the interesting structural features of glycoform V derivatives with a third Kdo is the concomitant truncation of the terminal disaccharide and the incorporation of P-EtN on the second Kdo with Rha on the third Kdo. The minimal LPS structure that can support incorporation of a third Kdo requires WaaO-mediated addition of glucose and hence serves as a branch point in determining switches between glycoform I and glycoform IV/V derivatives [62]. Primarily this switch is regulated by levels of WaaR and WaaZ, whose expression are regulated by induction of RpoE and PhoB/R TCS. Truncation of the terminal disaccharide suggested that the WaaR glycosyltransferase is limiting under RpoE-inducing conditions. This was indeed experimentally validated by observed repression of WaaR synthesis due to the RpoE-regulated RybB sRNA and to some extent by another RpoE-regulated sRNA MicA (Figure 2). At the same time, transcription of the *waaZ* gene is induced under such conditions. Physiologically, switch to the synthesis of glycoform derivatives with a truncation in the outer core, hence lacking terminal heptose, might be important for escaping detection by host, since such derivatives cannot incorporate *O*-antigen. Importance of the *O*-antigen incorporation can be critical, as it becomes essential for survival in *Salmonella* in the absence of the *rpoE* gene [78]. Thus, a coordinated molecular programming ensures increased synthesis of the WaaZ Kdo transferase under RpoE-inducing conditions, and the EptB P-EtN transferase, the induction of BasS/R-dependent WaaS synthesis with a simultaneous repression of WaaR and MgrR synthesis.

### 8.5. Transcriptional Regulation of Major LPS Biosynthetic Operons by RfaH

RfaH is a paralog of NusG family of universally conserved transcription factors. RfaH is unique in its specificity for recognition of only those operons that contain a short 8-nt conserved sequence (GGCGGTAG) in the 5′ UTR called the *ops* (operon polarity suppressor) pause site. Thus, RfaH regulates the expression of genes that play important functions in virulence such as the synthesis of LPS core, *O*-antigen, haemolysin, capsule and for conjugation [11,12]. Recruitment of RfaH prevents transcriptional termination and enhances transcriptional elongation of long *waaQ* and *rfb* operons. Consistent with a role for regulation of expression of *waaQ* and *rfb* operons, they contain an 8-nt conserved *ops* site and the JUMPstart site in their 5′ UTR and also lack a ribosome-binding site. In the absence of RfaH, LPS is truncated, since the expression of the *waaQ* operon is severely compromised and Δ*rfaH* mutants exhibit permeability defects, the induction of RpoE-dependent envelope stress response and are avirulent in pathogenic bacteria [11]. RfaH without interaction with the *ops* site exists in an inactive form with a closed conformation with its C-terminal domain (CTD) folded in an α helical hairpin tightly packed against its N-terminal domain (NTD). In this conformation, the NTD of RfaH is not accessible for DNA-RNA polymerase interaction [79]. However, upon encountering the *ops* site in the presence of RNA polymerase, the CTD of RfaH folds into a β-barrel allowing recruitment of S10 ribosomal protein to couple transcription with translation and also enhance transcriptional elongation. Recently, a new RirA sRNA (RfaH interacting RNA) was identified while analyzing factors that induce transcription of the *rpoE* gene [11]. Overexpression of the 73-nt RirA sRNA, located in the 5′ UTR of the *waaQ* operon, increases transcription from the *rpoE*P3 promoter that specifically responds to LPS defects. RirA overexpression abrogates the synthesis of *O*-antigen, causes reduction of LPS amounts and truncation in the LPS core, thus mimicking the Δ*rfaH* phenotype. Indeed, RirA directly interacts with RfaH in the presence of RNA polymerase and this interaction is dependent on the presence of the *ops* site within the *rirA* RNA [11]. This RirA-RfaH interaction could lead to loss of the specificity for recognition of *ops*-containing operons, like *waaQ* and *rfb* operons, needed for LPS core and *O*-antigen biosynthesis.

## 9. Signal Transduction in Response to LPS Defects

**Figure 3:**
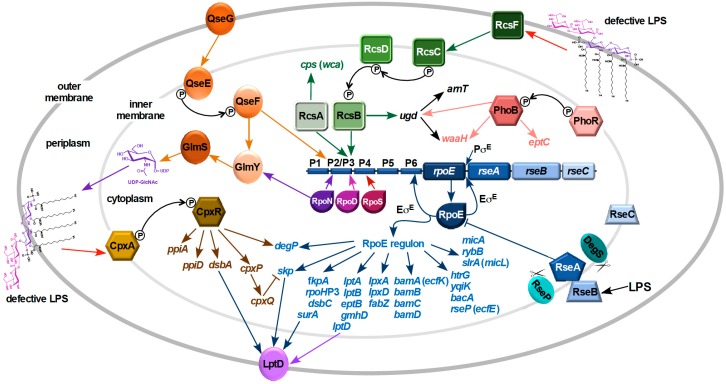
Pathways of sensing defects in LPS biosynthesis/assembly: sensing via the Rcs phosphorelay system, which leads to the induction of transcription from the sigma 70-regulated *rpoE*P3 promoter requiring RcsB as activator and the activation of Qse TCS induces the *rpoE*P2 promoter with QseF acting as an activator of RpoN. Severe defects in LPS induce the Rcs phosphorelay cascade leading to phosphorylation of RcsB. Phosphorylated RcsB acts as an activator for the transcription directed from the *rpoE*P3 promoter leading to the induction of RpoE-mediated stress response. The overlapping *rpoE*P2 promoter also responds to LPS defects albeit to a lower extent. Severe defects in LPS also induce the CpxR/A TCS. Eσ^E^ is required to maintain envelope integrity by regulating expression of genes whose products are involved in protein folding in the periplasm, assembly of OMPs (*bam* genes) and certain genes whose products are involved in LPS biosynthesis/modifications. Release of LPS from the Lpt system can lead to association of LPS with RseB, which relieves inhibition of RseA proteolysis and can lead to accumulation of free RpoE without RseA.

Integrity of the OM and homeostasis of various overall cell envelope components is critical for growth and the viability of bacteria. This requires correct assembly of OMPs, a balance between synthesis of peptidoglycan, phospholipids and LPS. This in bacteria requires function of various regulon members of RpoE and Cpx TCS. Severe defects in LPS biogenesis in response to underacylation, defects in LPS when the LPS assembly factor LapB is missing or depletion of Lpt translocation system, truncation in the inner core or when LPS synthesis is compromised particularly when bacteria synthesize minimal LPS derivatives like Kdo_2_-lipid A, Kdo_2_ lipid IV_A_ or only lipid IV_A_ derivatives, cause major cell envelope perturbations leading to a signal response that causes activation of the RpoE sigma factor, induction of the Rcs phosphorelay and activation of the Cpx TCS [7,9,10,11]. The extent of induction of these stress responses varies depending upon the severity of defects. Early work addressing activation of RpoE, when the OMP assembly is compromised, revealed that strains lacking GmhD (HtrM), which synthesize Kdo_2_-lipid A Re LPS, overexpress exopolysaccharide and have a constitutive elevation of RpoE response with a concomitant decrease in OMP content [17]. The exopolysaccharide synthesis is known to be positively regulated by the Rcs TCS by increasing transcription of *wca* genes [80]. Subsequently, during deciphering of the RpoE regulon, it was revealed that some of the genes involved in either LPS biosynthesis, LPS modifications, LPS transport and factors involved in OMP folding and insertion in the OM are part of this regulon [14]. In further studies, detailed mutational analysis of various genes, whose products are either involved in LPS biosynthesis or LPS modifications, coupled with mass spectrometric analysis of LPS of all such mutant derivatives and an impact on envelope stress response was undertaken in series of studies [7,9,11]. These studies reveal a full circuit wherein defects in LPS cause a major induction of RpoE and RpoE induction without any mutations in LPS biosynthetic/assembly pathways lead to several alterations leading to the accumulation of specific LPS glycoforms and various modifications (Figure 3). Some of the major conclusions and major pathways of stress response are discussed here. These studies showed that underacylation of LPS, when the core biosynthetic pathway is intact, does not cause any major induction of either RpoE or Cpx systems [7]. Only when all three acyltransferases are deleted, as in a strain Δ(*lpxL lpxM lpxP*), a modest induction of RpoE-dependent pathway is induced. In contrast, Δ*waaC* strains synthesizing Kdo_2_-lipid A LPS exhibit a nearly 3-fold induction of RpoE pathway without impacting the Cpx system. However, tetraacylated derivatives synthesizing Kdo_2_-lipid IV_A_ like Δ(*waaC lpxL*) or Δ(*waaC lpxL lpxM lpxP*) exhibit a 3-4-fold induction of both RpoE and Cpx pathways even under permissive growth conditions. Consistent with these are the findings that Δ*waaA* mutants synthesizing lipid IV_A_ precursor species exhibit maximal induction of RpoE and Cpx pathways. Given much higher induction of RpoE in Δ*waaC* mutants and lipid IV_A_ synthesizing strains, as compared to its modest induction in Δ(*lpxL lpxM lpxP*) mutants, suggests an acute requirement for the incorporation of Kdo and initial steps in LPS core biosynthesis for cell envelope integrity. Interestingly, a Δ(*lpxL lpxM lpxP*) strain was found to synthesize LPS primarily of glycoform with a third Kdo residue and a truncation of outer core disaccharide, when grown in lipid A modifying conditions [7]. Furthermore, mutations in some of the conserved early glycosyltransferases encoding genes, like *waaF*, *waaG*, *waaO* or *waaP* whose product phosphorylates Heptose I, or in the absence of RfaH transcriptional factor whose product is required to ensure transcription/translation and prevent premature termination of transcription of the *waaQ* operon also induce the *rpoE* transcription [11].

Consistent with sensing of correct assembly of LPS by RpoE, such a phenotype was used to identify LapA and LapB assembly factors, since the lack of LapB causes increased constitutive induction of RpoE and Cpx regulons quite like Δ*waaA* mutants [9]. As mentioned above, Δ(*lapA lapB*) mutants produce more LPS due to stabilization of LpxC, accumulate precursor species of LPS with incomplete core, defects in acylation of lipid A and reduced amounts of LptD in the OM. Thus, given the overwhelming evidence, it is fair to conclude that severe defects in either early steps in LPS core biogenesis or when LPS is composed of only Kdo_2_-lipid IV_A_ or when LPS assembly is defective lead to the induction of RpoE envelope stress response.

## 10. Mechanism of Signal Transduction

Here, we will discuss the activation of RpoE regulon in response to LPS defects with an overlap in the activation of the Rcs phosphorelay system, which jointly respond to LPS defects (Figure 3). We do not know how the Cpx system senses LPS defects as it can respond to several envelope stress signals in the IM, periplasm and OM. One of the main observations has been that only those structural LPS changes/defects that induce the Rcs system also induce RpoE. Thus, induction of PhoB/R-dependent GlcUA incorporation, BasS/R-dependent lipid A modifications, lack of either WaaY, or WaaZ or WaaS or truncation of outer core neither induce the Rcs system nor the RpoE-regulated stress response [11,55]. In a comprehensive study, a systematic approach of dissection of the transcriptional regulation of the *rpoE* gene and stimuli that induce or repress was taken based on previously observed RpoE induction in Δ*waaA* or strains synthesizing Kdo_2_-lipid IV_A_ derivatives [7,11]. When the *rpoE* gene was initially identified, two complementary studies showed that transcription of the *rpoE* gene is positively autoregulated from one of its promoters since it is recognized by Eσ^E^ polymerase and its activity is induced when OMP maturation is dysfunctional or due to an imbalance in OMP expression [81,82]. However, the regulation of distal promoter or promoter region (previously called the *rpoE*P1 promoter) remained elusive. Besides positive transcriptional regulation, RpoE is negatively regulated by RseA and RseB proteins, whose encoding genes are co-transcribed with the *rpoE* gene [83]. The RseA inner membrane protein associates via its cytoplasmic N-terminal domain with RpoE, thereby sequestering RpoE in the IM under non-stress conditions [83]. This inhibitory effect of RseA is relieved upon OMP misfolding or overexpression of some OMPs by sequential degradation of RseA initiated by DegS in the periplasm, followed by the second site inner membrane proteolysis by RseP (EcfE) and subsequent degradation of the N-terminal RseA in the cytoplasm [84]. The periplasmic C-terminal domain of RseA associates with RseB and prevents RseA degradation. Since the autoregulated *rpoE* promoter (P6 promoter), previously called *rpoE*P2 promoter, responds to effective concentration of available RpoE that complexes with core RNA polymerase, its activity reflects both transcriptional increase in the *rpoE* expression and its release from RseA and hence its activity or activity of reporter promoters of its regulon members (*rpoH*P3) does not directly reflect response to LPS defects.

During the studies aimed to understand the mechanism of *rpoE* induction in response to LPS defects and examination of transcriptional regulation of upstream element, it turned out that the *rpoE* gene is transcribed from six promoters and, among these, activity of *rpoE*P2 and *rpoE*P3 promoters is specifically induced when LPS is defective (Figure 3). Among these, transcription from the *rpoE*P3 promoter is significantly induced when either the LPS core biogenesis is defective or when LPS assembly is compromised or by challenge with LPS-binding antibiotic like polymyxin B or treatment with agents like AMV that induce non-specifically different TCS [11]. Global mutational screen identified insertion/deletion mutations mapping to genes involved in the early steps of LPS core biosynthesis such as *waaC*, *waaF*, *waaG*, *waaO* and *waaP*. Among these, maximal 7-fold induction of the *rpoE*P3 promoter was observed in Δ*waaC* mutants synthesizing Kdo_2_-lipid IV_A_ [11]. This activation was nearly abrogated in Δ(*waaC rcsB*) mutants suggesting that the signal of activation due to defective LPS requires the RcsB response regulator [11]. Interestingly, mutations in either *waaC* or *waaF* or *waaP* exhibit deep-rough phenotype accompanied by the induction of Rcs-regulated exopolysaccharide synthesis. Analysis of various other response regulators did not show any direct participation [11]. The activity of the same *rpoE*P3 promoter was found to be induced nearly 3–4 fold in Δ*rfaH* background and by the overexpression of *rirA* sRNA [11]. The 73 nt RirA sRNA contains *ops* element, located in the 5′ UTR of *waaQ* operon and acts by decreasing the RfaH activity, by directly binding to RfaH in the presence of RNA polymerase. The overexpression of *rirA* causes a reduction in the expression of operons that require the RfaH activity. When RirA is in excess, it causes a decrease in the expression of *waaQ* operon members leading to truncation of the LPS core and abolishes the incorporation of *O*-antigen, explaining the specific induction of LPS responsive *rpoE*P3 promoter [11]. Additional evidence that the *rpoE*P3 promoter responds to LPS-specific alterations is based on an increase in transcription from this promoter when bacteria are challenged in early growth phase by either polymyxin B or AMV. The induction of RpoE regulon concomitant with Rcs pathway activation is also observed when any component of Lpt is depleted, since under such conditions LPS is modified by the Rcs-regulated colanic acid exopolysaccharide [85].

A direct proof that the *rpoE*P3 promoter is positively regulated by the Rcs system comes from in vitro DNA binding assays with purified RcsB, establishing the presence of conserved RcsB motif in the *rpoE*P3 promoter region [11]. This was supported by deletion and mutational analysis of RcsB-binding motif. Run-off assays established the authenticity of recognition by the house-keeping sigma factor and mutational analysis reinforced that RcsB acts as a positive regulator in response to LPS defects. Transcription from the *rpoE*P3 promoter is also activated in Δ(*lapA-lapB*) which also requires RcsB. It should be noted that the RpoN-regulated *rpoE*P2 promoter also responds to LPS defects albeit to a much-reduced extent (only a 70% to 2-fold increase as compared to the 7-fold induction by Δ*waaC*) and that too mainly upon entry into the stationary phase. Since the *rpoE*P2 promoter requires QseF as an activator [11], it will be interesting to know how the Qse TCS senses LPS.

A role for sensing of LPS defects by the Rcs signalling pathway was proposed in earlier studies by examining a mutant strain carrying *rfa-1* mutation. Such a strain synthesizes LPS composed of only two heptose residues lacking any glucose in the core region and exhibits induction of *cps* genes leading to overexpression of exopolysaccharide [80]. Furthermore, it was shown that this LPS defect requires the outer membrane lipoprotein RcsF, since induction of cps transcription in *rfa-1* mutant was abolished in Δ*rcsF* mutants [80]. Interestingly, overexpression of *cps* genes due to LPS defects did not occur due to the increased *rcsF* transcription, rather could be the consequence of other mechanisms of the RcsF activation. More recent studies on RcsF revealed that it exists in complex with many OMPs [86] and such RcsF complex uses its positively charged, surface-exposed N-terminal domain to detect alterations in lateral interactions between LPS molecules (changes in phosphorylation status of the LPS core), which lead to the induction of Rcs system. This activation of Rcs pathway in turn can activate RcsB to induce the *rpoE*P3 promoter when LPS defects are encountered (Figure 3).

Another model postulates that the periplasmic RseB binds to LPS that might be released in an off pathway during translocation via the Lpt system [84]. Usually, RseB association with RseA inhibits its proteolysis. However, LPS binding to RseB relives inhibition of proteolysis by disassociation of the RseA-RseB complex (Figure 3). In support of this model, authors show that RseA-RseB dissociation by LPS is inhibited in the presence of LptA, suggesting that the periplasmic accumulation of LPS, due to release from Lpt proteins in the periplasmic bridge, can promote the dissociation of RseB from RseA and cause RpoE induction. However, the validity of this model needs further studies as free LPS is not known to accumulate when the LPS core is truncated and severe defects in LPS, particularly upon accumulation in the IM when the Lpt system is depleted, also induce RpoE. Analysis of distribution of free RpoE vs. RpoE bound to the IM in Δ*waaA* has revealed that the elevated RpoE activity can be attributed to increased release of RpoE from the IM in such mutants [87]. However, a joint signaling when LPS is defective via the Rcs system and RseB can amplify RpoE induction and hence these models are not mutually exclusive.

In summary, comprehensive analysis of various LPS defects has revealed that RpoE activity is specifically induced when LPS biosynthesis or assembly is compromised by the activation of the *rpoE*P3 promoter with phosphorylated RcsB acting as an activator and the sensing of LPS defects occurs via the Rcs phosphorelay system (Figure 3).

## 11. Conclusions

The LPS synthesis is subjected to a tight regulation at variety of steps. Regulation of LpxC amounts by FtsH and LapB constitutes an essential step to maintain a balanced synthesis of LPS and phospholipids. Further, MsbA by preferential selectivity of hexaacylated LPS provides an early checkpoint, preventing transport of precursor underacylated LPS species. LapB recruits LpxC, FtsH, glycosyltransferases, myristoyltrasferase LpxM and other proteins that are required for LPS synthesis at the IM, presumably acting as a scaffold to deliver completely synthesized LPS to its other interacting partners in the Lpt transport system. LPS is heterogeneous in composition and its composition is dynamically altered by a host of factors. These LPS alterations are regulated by the RpoE sigma factor, TCS like BasS/R, PhoP/Q, PhoB/R and Rcs system with an overwhelming role played by noncoding sRNAs. The Rcs system senses LPS defects resulting into phosphorylation of RcsB, which leads to activation of transcription from *rpoE*P3 promoter leading to RpoE induction. Finally, the transenvelope Lpt complex of seven essential proteins acts as a single unit and in an ATP-dependent manner ferries LPS across the periplasmic bridge comprised of LptA for delivery to the OM component of LptD/E in a continuous manner. Although tremendous progress has been achieved in the recent past, it is still unknown how the LptB cytoplasmic component of Lpt system is localized to the IM and how the proteolytic activity of FtsH is regulated by LapB protein and other factors. Further studies are also needed to demonstrate that LapB acts as the scaffold for LPS assembly and its coordination with the Lpt transport system, although LapA/LapB proteins have been demonstrated to co-purify with Lpt components. As LPS is essential, its essential steps in biosynthetic pathway and Lpt transport have been validated targets of discovery of new antibiotics. Already inhibitors of LpxC and a peptidomimetic POL7080 based on the cationic antimicrobial peptide that inhibits the LptD activity are in advanced clinical trails and many additional antimicrobial compounds using LPS synthesis/assembly are bound to be discovered in future.

## Abbreviations

AMV	ammonium metavanadate
GlcUA	glucuronic acid
IM	inner membrane
Kdo	3-deoxy-α-d-*manno*-oct-2-ulsonic acid
l-Ara4N	4-amino-4-deoxy-l-arabinose
LPS	lipopolysaccharide
OM	outer membrane
OMP	outer membrane protein
*ops*	operon polarity suppressor
P-EtN	phosphoethanolamine
sRNA	noncoding small regulatory RNA
TCS	two-component system
TPR	tetratricopeptide repeats
UTR	untranslated region
